# Genetic Mapping and QTL Analysis of Stigma Color in Melon (*Cucumis melo* L.)

**DOI:** 10.3389/fpls.2022.865082

**Published:** 2022-05-09

**Authors:** Yuanzuo Lv, Peng Gao, Shi Liu, Xufeng Fang, Taifeng Zhang, Tai Liu, Sikandar Amanullah, Xinying Wang, Feishi Luan

**Affiliations:** ^1^Key Laboratory of Biology and Genetic Improvement of Horticulture Crops (Northeast Region), Ministry of Agriculture and Rural Affairs, Northeast Agricultural University, Harbin, China; ^2^College of Horticulture and Landscape Architecture, Northeast Agricultural University, Harbin, China

**Keywords:** melon, stigma color, gene mapping, CAPS markers, chlorophyll

## Abstract

Melon is an important Cucurbitaceae crop. Field observations had shown that the green stigmas of melon are more attractive to pollinators than yellow stigmas. In this study, F_2_ and F_2:3_ populations obtained by crossing MR-1 (green stigma) and M4-7 (yellow stigma) were used for genetic analysis and mapping. A genetic map of 1,802.49 cm was constructed with 116 cleaved amplified polymorphism sequence (CAPS) markers. Two stable quantitative trait loci (QTLs) linked to the trait of stigma color were identified on chromosomes 2 (*SC2.1*) and 8 (*SC8.1*), respectively. An expanded F_2_ population was used to narrow down the confidence regions of *SC2.1* and *SC8.1*. As a result, *SC2.1* was further mapped to a 3.6 cm region between CAPS markers S2M3 and S2B1-3, explaining 9.40% phenotypic variation. *SC8.1* was mapped to a 3.7-cm region between CAPS markers S8E7 and S8H-1, explaining 25.92% phenotypic variation. This study broadens our understanding of the mechanisms of stigma color regulation and will be of benefit to the breeding of melon.

## Introduction

The economic benefits of pollination are the primary determinants for fruits, vegetables, and seed production, which influence at least 87 leading food crops around the world (Kevan and Viana, 2003; Klein et al., 2007). Pollination is crucial for reproduction in flowering plants whereby the male gamete (pollen grains) from the anther comes into contact with the female gamete (stigma). Likewise, successful reproduction is highly dependent on efficient pollinators (Blacquiere, 2010).

The trichromatic vision of bees is known to effectively handle different photoreceptor classes (blue, green, and UV ranges) (Peitsch et al., 1992). The floral pattern at the center of a flower can guide pollinators in finding the nectar (Lunau et al., 1996; Dafni and Giurfa, 1999; Lunau, 2005; Davies et al., 2012). Therefore, the center of a flower is easily recognizable by bees (Biesmeijer et al., 2005; Davies et al., 2012).

There is an important feature of distinct stigma color in each female flower, which directly provides a great support in plant reproduction. However, stigma color is a neglected trait in many cultivars of different fruit crops but it significantly possesses enormous benefits for desired crop production using different molecular breeding approaches. It has been reported that the level of carotenoid biosynthetic genes is associated with the accumulation of carotenoids and the resultant stigma color in *Crocus sativus* (Ahrazem et al., 2019). Similarly, during the transition from yellow undeveloped to red fully developed stigmas, the accumulation of zeaxanthin occurred due to the expressions of *CsPSY* and *CsLcyb* (Castillo et al., 2005). In a stable inherited yellow stigma tomato mutant (*ys*) that was obtained using ethyl methane sulfonate, a single recessive gene was found to regulate the yellowing of stigma due to the accumulation of naringenin chalcone in *ys* (Zhao et al., 2017). In rice, two genes controlling the purple stigma were mapped on chromosomes 1 and 6 using 1,300 F_2_ populations that are derived from XQZ (purple stigma and red lemma tip) and Kitaake (white stigma and colorless lemma tip) (Wang, 2016).

Melon (*Cucumis melo* L., 2n = 24) is an attractive fruit crop due to the extreme divergences in phenotypic diversity with a reported production of more than 42 million tons globally in 2020 (FAOSTAT; http://faostat.fao.org). Melon flowers bear yellow petals and yellow to green stigmas, which are mostly pollinated by bees under the natural field conditions (Rodrigo Gomez et al., 2016). Therefore, the green stigma can be favorable over the yellow stigma for the production of desired fruits and seeds, aimed at different breeding purposes, respectively.

Compared to the traditional breeding approaches, the marker-assisted selection (MAS) system has been proved as a more effective strategy that is used for genetic mapping of different crop traits. Until now, not all the genetic components of melon cultivars have been dissected, and there is a dire need to investigate the genetic patterns and molecular mechanisms associated with desirable traits, which should be an important part of breeding programs, aimed at genetic improvement of crops. Therefore, this study was aimed at identifying the stable quantitative trait loci (QTLs) regulating the stigma color, using the respective mapping population of F_2_ and F_2:3_ families that are derived from the crossing of MR-1 (green stigma) and M4-7 (yellow stigma) melon lines, respectively. The present novel outcomes would be beneficial to provide the fundamental basis for the genetic understanding of melon stigma color trait.

## Plant Materials

**Figure 1:**
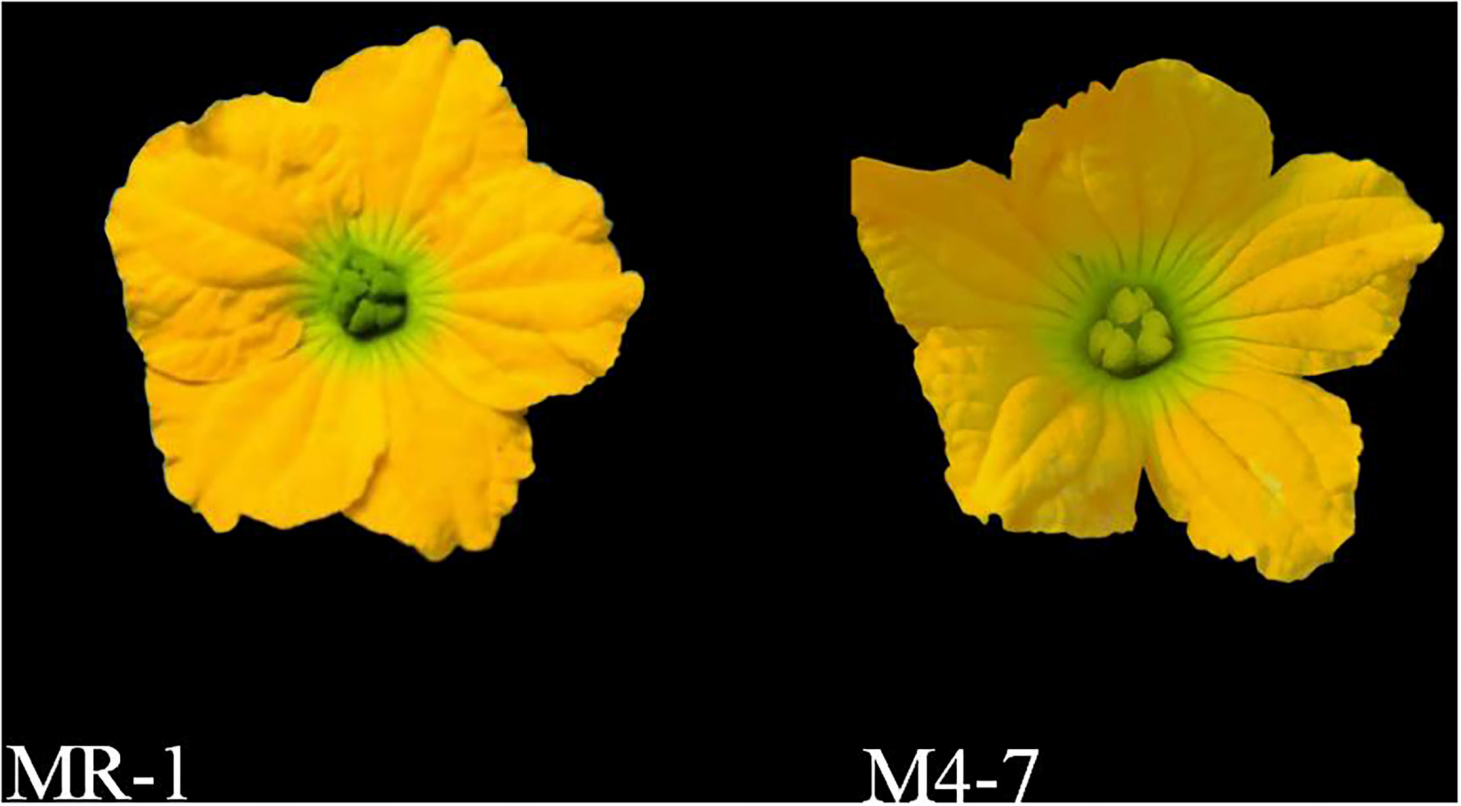
The primary phenotypes of MR-1 and M4-7.

Two different parent lines of melon MR-1 (P_1_, female with green stigma) and M4-7 (P_2_, male with yellow stigma) were selected as experimental material (Figure 1) and crossed to produce their F_1_ progeny. The field experiments were performed in the plastic greenhouse at Xiang Yang Experiment Agricultural Station, Northeast Agricultural University, Harbin, China (lat. 44°04′N, long. 125°420′E) over 3 years (from 2019 to 2021). The plants were grown using a completely randomized design (CRD), and standard horticultural practices were adopted for successful germination.

In 2019, an F_2_ mapping population comprising 133 plants was obtained from the parental lines crossing, and 55 plants with extremely divergent phenotypes were self-crossed to get the respective F_2:3_ families. In 2020, all of the 55-F_2:3_ families (20 plants for each family and a total of 1,100 individuals) were planted to detect the stable QTLs associated with stigma color. In 2021, an expanded population of 545-F_2_ lines was also planted. In this study, fifteen (15) plants, each line for P_1_, P_2_, and F_1_, generations, were grown along with all generations and subsequently utilized for genetic linkage analysis of melon stigma color.

## Observation of Flower-Visiting Insects

The observation site was located in Harbin, Heilongjiang Province, China. The numbers of flower-visiting insects on the stigmas of both parent lines “MR-1 and M4-7” were visually observed during the peak flowering. The flowers were observed on daily basis from 7:30 up to 9:30 am and photos were taken for 3 consecutive days using a Gopro7 camera in the time-lapse mode.

## Phenotypic Data Collection

The stigmas of respective flowers of F_2_ and F_2:3_ mapping populations were checked, three stigmas from each plant were chosen, and color phenotypes were collected from three flower repetitions using a 3NQ portable colorimeter (NR10QC). The respective color hues of “L,” “A,” and “B” were recorded, where “L” indicated lightness, “A” indicates red-green difference, “B” indicates yellow-blue difference, and “E” denoted the net color difference. The final three-dimensional orthogonal graph was plotted using the color phenotypes, and color difference “E” was calculated for each plant according to the following equation:


$$\begin{array}{l} E={\left(L^{2}+A^{2}+B^{2}\right)}^{\wedge }\left(1/2\right) \end{array}$$

## Whole Genome Re-sequencing and Markers Development

The leaf material (0.2 g) was taken from 2-week-old seedlings of P_1_, P_2_, F_1_, and F_2_ progeny, chilled in cryogenic liquid nitrogen, and freshly stored at −80°C. Then, high-quality DNA was isolated using the cetyl trimethyl ammonium bromide (CTAB) protocol with slight modifications. The quantified DNA was identified with 1% agarose gels electrophoresis fragmented with Bioruptor (Thermo Fisher Scientific, USA), and 200–300 bp fragments were prepared for the High-throughput Illumina™ X10 sequencing platform. The resultant clean end bases of paired-end sequencing of both parent lines were aligned to *de novo* assembled reference genome (DHL92, v3.5.1) of melon using the default algorithm of Burrows-Wheeler Aligner (BWA)-Maximal Exact Match (MEM) (https://sourceforge.net/projects/bio-bwa/files/). The Binary Alignment/Map (BAM) files were similarly used for the filtering and alignment of re-sequenced clean end bases. The major single-nucleotide polymorphisms (SNPs) were obtained and annotated using the SnpEff tool (v4.3), then 500 bp flanking sequences were extracted from each random SNP site.

The cleaved amplified polymorphism sequence (CAPS) markers were developed by identifying the suitable SNP-based restriction endonucleases (*Eco*RI, *Hin*dIII, *Pst*I, *Bam*HI, and *Bcl*I) using SNP2CAPS v0.6 and primer premier v6, respectively (Amanullah et al., 2020). The PCR products of each CAPS marker were digested with restriction endonucleases to verify the codominant polymorphism of each marker. The PCR reaction mixture was prepared as follows: a pair of primers (8–10 pmol), deoxynucleotide triphosphates (dNTPs) (0.25 mM), Taq buffer (10×), and Taq polymerase (1 unit). A thermocycler PCR was performed by preheating the samples at 94°C for 7 min, followed by 30 cycles of 30 s at 94°C, 30 s at 60°C for the first cycle, and a stepwise down-gradient of 0.5°C per cycle, followed by 60 s at 72°C. Then, 10 cycles of 30 s at 94°C, 30 s at 45°C, and 60 s at 72°C were performed, followed by elongation for 5 min at 72°C. For enzyme digestion, a reaction mixture consisted of 4 μl of PCR product, 4.8 μl of ddH_2_O, 1 μl of CutSmart buffer, and 0.2 μl of restriction enzymes and was incubated at 37°C for 4 h. The digested products were subsequently cleaved by 1% agarose gel electrophoresis.

## Linkage Mapping and QTL Analysis

A genetic linkage map was developed and QTLs were mapped by using the default parameter settings of the IciMapping (v4.2) tool as followed (Meng et al., 2015). All the genotyped CAPS markers were evenly scattered across the whole genome chromosomes. The coded genotypic data of respective F_2_ mapping populations were grouped and anchored over the whole genome chromosomes according to their exact physical positions and maximum likelihood means. The default chi-squared method and Kosambi's mapping function were selected to determine the segregation ratio of genotypic markers and to estimate the genetic distances, intervals, and positions at *p* (>0.001). The significant QTLs were defined above the threshold level of the default logarithm of odd (LOD) score (3.00) and the genome-wide type I error at α = 0.05.

## qRT-PCR Analysis

Total RNAs were extracted using DiNing DP230-01 Plant Total RNA Purification Kit following the manufacturer's protocol (DiNing Biotech, Beijing, China). The qRT-PCR was performed on RNA extracted from the stigma of MR-1 and M4-7 on the day the female flowers were opened using the *Actin* gene as *MELO03C023264*. For qRT-PCR, assays were prepared using 20 ng cDNA and 300 nM of each primer in a 10 μl of reaction mixture with the addition of SYBR Green I Master Mix. qRT-PCR was performed using three biological replicates for each tissue sample and at least three technical replicates of each biological replicate. After normalization of the transcript level of each gene with the most suitable internal control gene for each sample, fold change was calculated by a 2^−ΔΔCT^ method.

## Results

### Pollinator Observation

We observed that some flower-visiting insects generally fed on flowers for pollen and nectar, while some insects only stayed on flowers with no further activity (Figure 1). The most frequent pollinators were *Apidae* (bees), followed by *Muscidae* (flies). The proportion of visiting pollinators for green stigma melon (MR-1) was three times more than that of yellow stigma melon (M4-7) (Table 1).

**Table 1:** Visitation of flower visiting insects in melons with different stigma colors.

**Sample**	**Time**	**Number of flower-visiting insects**	**Ratio of Hymenoptera**	**Ratio of Diptera**
MR-1	2 h/d	22.1 ± 3.1	78.90%	10.06%
M4-7		6.80 ± 1.4	80.30%	11.50%

### Genetic Analysis of Melon Stigma Color

**Figure 2:**
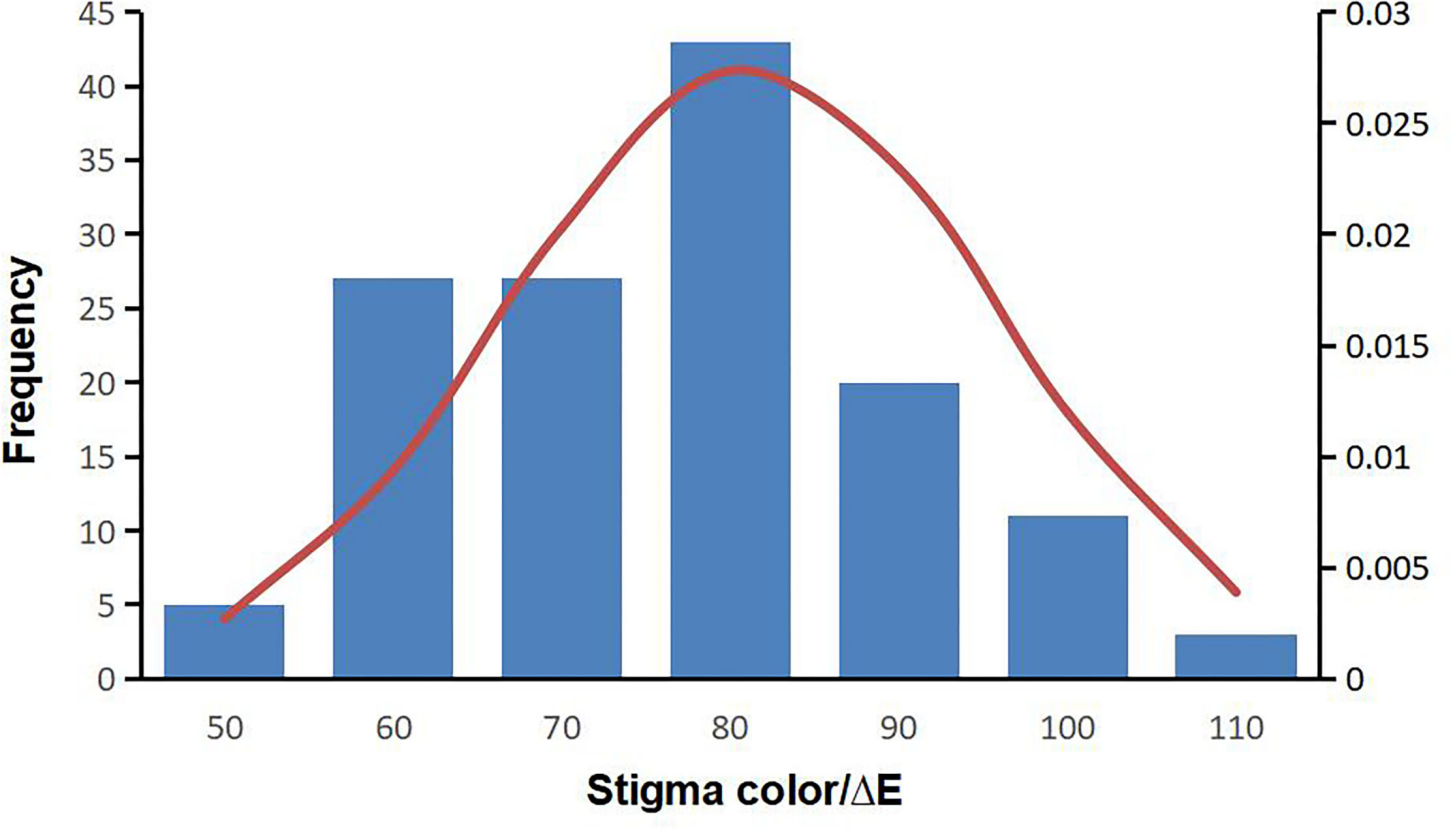
Frequency histogram of stigma E value of the F_2_ population in 2019.

“Lab” color space is based on the human eye's perception of color and could represent the colors observed by the human eye. The “E” values of MR-1 and M4-7 stigmas were measured for 3 consecutive years and the data obtained were stable, with the “E” value of M4-7 being consistently higher than that of MR-1 (Supplementary Table S1). According to Figure 2, the “E” value displays continuous variation among F_2_ individuals and follows a normal distribution, suggesting that melon stigma color is controlled by quantitative loci. Besides, there is no color difference between the stigmas in one plant individual.

### Linkage Map Construction and Primary Mapping

**Figure 3:**
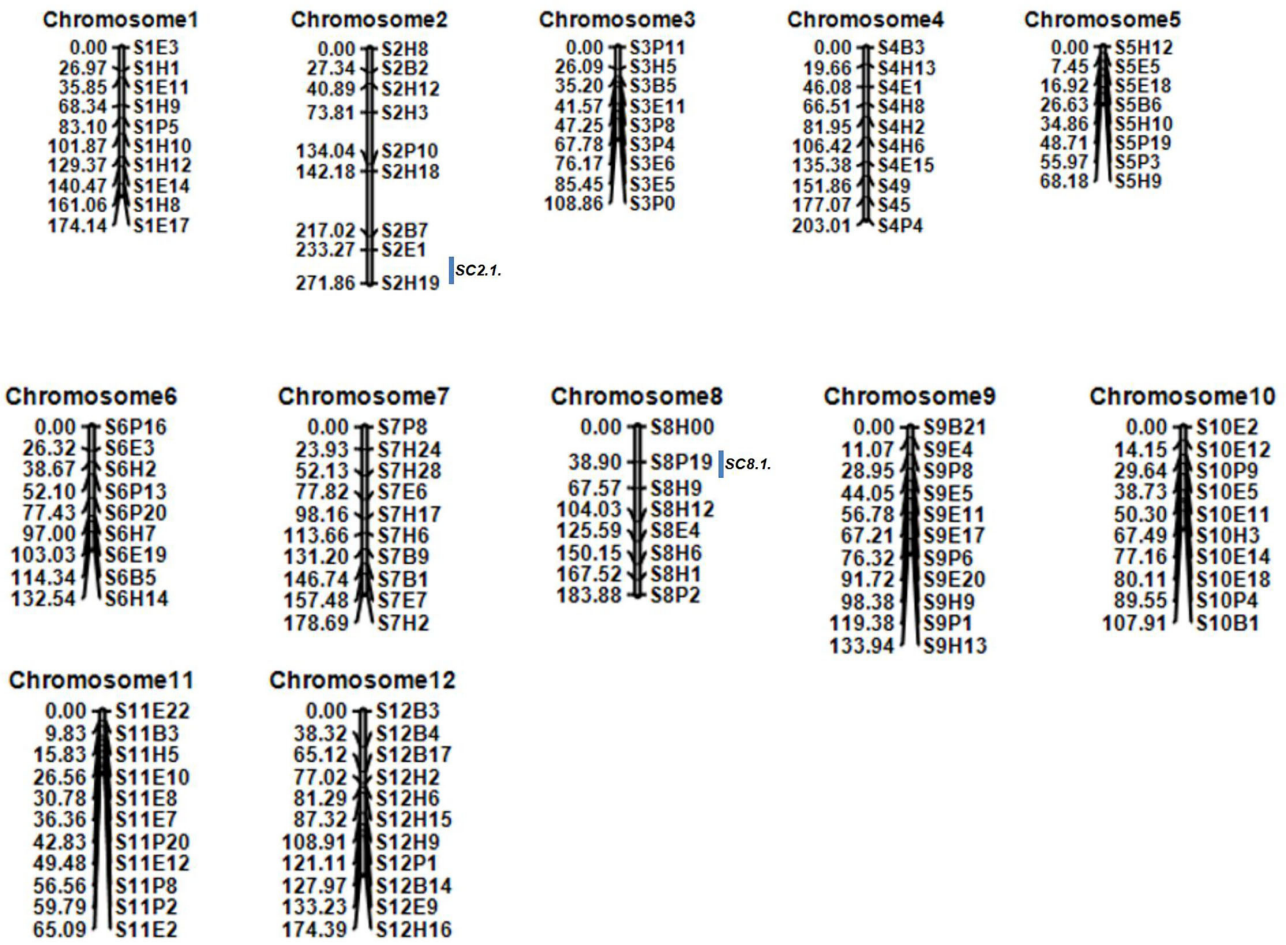
A constructed linkage map of melon chromosomes based on the F_2_ mapping population of a cross between parental lines “MR-1 and M4-7.” The box to the right of the chromosomes indicates the stable quantitative trait loci (QTL) identified as associated with stigma color by 2 years of data.

In 2019, a total of 116 CAPS markers were effectively developed and genotyped for linkage mapping, which spanned a total of 1802.49 cm length with an average of 15.54 cm over the whole genome chromosomes (Figure 3). The chromosome 8 showed the least number of CAPS markers and total of 8 polymorphic markers covered 183.88 cm length and separated with an average genetic distance of 8.32 cm; however, chromosome 12 exhibited more CAPS markers comprising 12 CAPS markers, which covered 174 cm length with an average distance of 14.06 cm between each marker.

Quantitative trait locus analysis exposed that three QTLs, *SC2.1, SC5.1*, and *SC8.1*, were mapped on chromosomes 2, 5, and 8, respectively (Table 2). *SC2.1* was mapped on the genetic position of chromosome 2 between flanking markers S2E1 and S2H19 with 5.92% phenotypic variance explained (PVE) and LOD value of 5.54, *SC5.1* was located on chromosome 5 between adjacent markers S5H10 and S5P3 with 12.30% PVE and LOD value of 3.90, and *SC8.1* was mapped on chromosome 8 between S8P19 and S8H9 with 23.18% PVE and LOD value of 14.29, respectively.

**Table 2:** Quantitative traits locus (QTL) analysis of melon stigma color using F_2_ population and F_2:3_ population, respectively.

**Year**	**Population**	**QTL name**	**Trait**	**Adjacent marker**	**Chr**.	**LOD**	***R* ^2^**
2019	F_2_	*SC2.1*	Stigma color/**Δ**E	S2E1~S2H19	2	5.54	5.92%
		*SC5.1*		S5H10~S5P3	5	3.91	12.30%
		*SC8.1*		S8P9~S8H9	8	14.29	23.18%
2020	F_2:3_	*SC2.1*		S2E1~S2H19	2	3.62	9.71%
		*SC8.1*		S8P19~S8H9	8	6.97	21.74%

In 2020, the F_2:3_ lines were generated from the same F_2_ self-crosses and the genetic map was re-constructed to verify the stable QTLs. Then, two stable QTLs, *SC2.1* and *SC8.1*, were found to be consistent with the F_2_ mapping population (Table 2); *SC2.1* explained somewhat lower phenotypic variation (9.71%) with an LOD value of 3.6 and was positioned between markers S2E1 and S2H19. Meanwhile, *SC8.1* explained a high phenotypic variation (21.74%) with the LOD value of 6.97 between markers S8P19 and S8H9, respectively.

**Figure 4:**
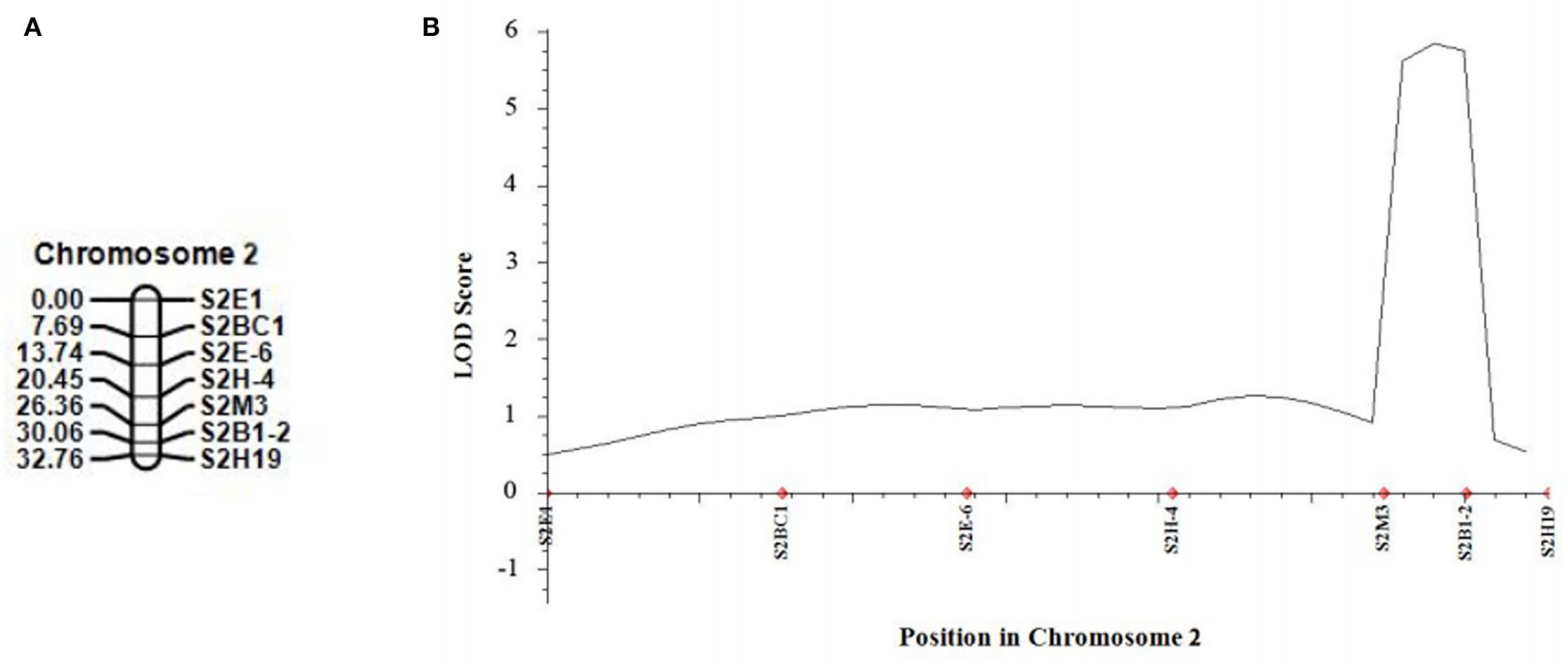
Validation of the stigma color quantitative trait loci (QTL) in melon. **(A)** Linkage map of melon chromosome 2, based on 540 F_2_ individuals derived from a cross between MR-1 and M4-7. **(B)** QTL curve for melon stigma color.

**Figure 5:**
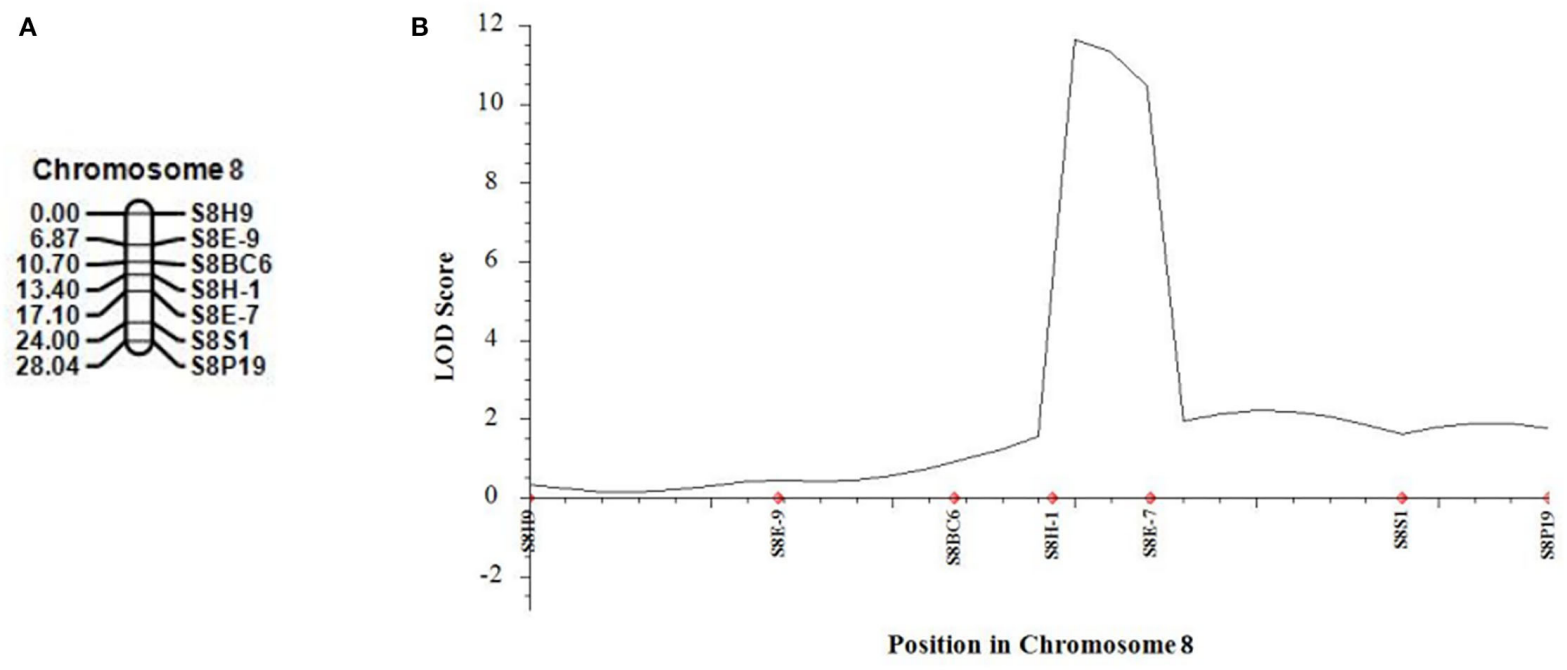
Validation of the stigma color quantitative trait loci (QTLs) in melon. **(A)** Linkage map of melon chromosome 8 based on 540 F_2_ individuals derived from parental lines crossing. **(B)** QTL curve for melon stigma color.

In 2021, we developed five new CAPS markers in the detected QTL regions (Table 3) and further validated that both candidate QTLs, *SC2.1* and *SC8.1*, were associated with stigma color. For this purpose, phenotypic data of an expanded mapping population comprising 545 F_2_ individuals were incorporated for QTL analysis (Table 4). The confidence region of *SC2.1* explained 9.40% phenotypic variation and further delimited to 3.6 cm and physical position from 24,730,977 to 25,015,025 bp exhibited 180 kb interval between flanking markers S2M3 and S2B1-2 (Figure 4). The confidence interval (CI) of *SC8.1* explained 25.92% phenotypic variation in the enlarged mapping population, further narrowed down to a 3.7 cm region, which corresponded to a 138 kb interval (from 30,395,830 to 30,532,659 bp) between markers S8H-1 and S8E-7 (Figure 5), respectively.

**Table 3:** Detailed information of the new polymorphic cleaved amplified polymorphism sequence (CAPS) markers within *SC2.1* and *SC8.1*.

**Marker**	**Sequence**	**Enzyme**	**Annealing temp**
S2BC1	F:GCAACAACCAATATCACACCAT	BamI	55
	R:GCTCAGAGGCTAGAGATTATTCAA		
S2E-6	F:GTGTAAGTAAGAGATTGATGAGAGG	EcoRI	55
	R:TGGTTACCAACTCGAAGCTAA		
S2H-4	F:TGTACCTCTGTAATTCTTCGGATG	HindIII	55
	R:TGTAACAACCCACACAAACTCA		
S2M3	F:CTTCTTCTATGATGGCTACAGTCTT	PstI	55
	R:CGAGATGGTTGCTATCCTTGG		
S2B1-2	F:TGGACAACATGCACATTACACT	BclI	55
	R:AAGGTCGAAGATCATCTCCGTAT		
S8H9	F:TTTCAACCCACACTCTCATCTTC	HindIII	55
	R:CTCAATTATTTCCCTCTCCTACCC		
S8BC6	F:TGGTAAGAGTAGGACAACATATAGG	BclI	55
	R:GGAATATACGTTCACTCCATCAAC		
S8H-1	F:GCATGATAGTGATGTAGGTGAGAA	HindIII	55
	R:GTTCGGAATGGGAAAGAAGGTT		
S8E-1	F:AGAAGGAGATGAATCAAGTCTA	EcoRI	55
	R:TGTACCATACGCAATCGTTAGTCG		
S8S1	F:CCGTTCATCACACTCCACAAG	PstI	55
	R:GCGAAGTAATCCTATAACAGTCATC		

**Table 4:** Quantitative traits locus (QTL) analysis of melon stigma color using expanded F_2_ population.

**QTL name**	**Trait**	**Adjacent marker**	**Marker interval position/cM**	**Chr**.	**LOD**	***R* ^2^**
*SC2.1*	stigma color/**Δ**E	S2M3~S2B1-2	3.6	2	5.86	9.4%
*SC8.1*		S8E-7~S8H-1	3.7	8	11.65	25.92%

**Figure 6:**
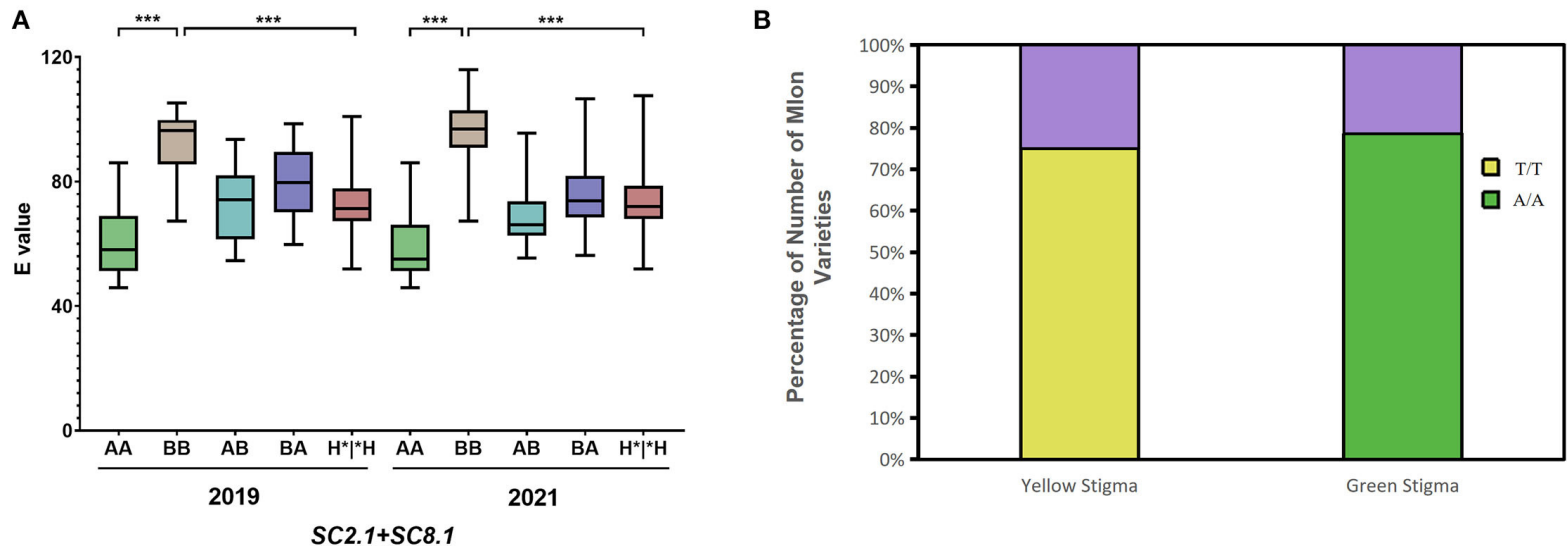
Genotype analysis of quantitative trait locus (QTL) and candidate gene (*MELO03C003165*). **(A)** The combined genotype and phenotype of *SC2.1*+*SC8.1* in F_2_ individuals were recorded in 2019 and 2021. In the horizontal coordinates, the former genotype belongs to *SC2.1*, the latter genotype belongs to *SC8.1*, and H*|*H represents the genotype containing H. **(B)** Relationship between genotype and stigma color of the *MELO03C003165* alleles in 110 melon varieties. The purple color shows the proportion of genotype and phenotype inconsistencies, Yellow and green show the proportion of genotype and phenotype concordance.

A pairwise combination of the genotypes of *SC2.1* and *SC8.1* in the F_2_ population was analyzed in 2019 and 2021 (Figure 6A). The genotype of candidate QTLs *SC2.1* and *SC8.1* in MR-1 with green stigma was *AA* while for M4-7 with the yellow stigma it was *BB* (the genotypes of *SC2.1* and *SC8.1* being indicated by markers S2H9 and S8P19), respectively. The *AA* genotypes of *SC2.1*+*SC8.1* depicted that the “E” value was significantly lower as compared to *BB* genotypes, and the stigma color showed a natural tendency of green color. In contrast, the stigma color inclined to yellow when the genotype of *SC2.1*+*SC8.1* was *BB*; however, when the genotype of *SC2.1*+*SC8.1* was heterozygous, the “E” value was generally reported at the intermediate level. These findings support our claim that *SC2.1* and *SC8.1* jointly affected melon stigma color.

### Analysis of Predicted Candidate Genes

**Figure 7:**
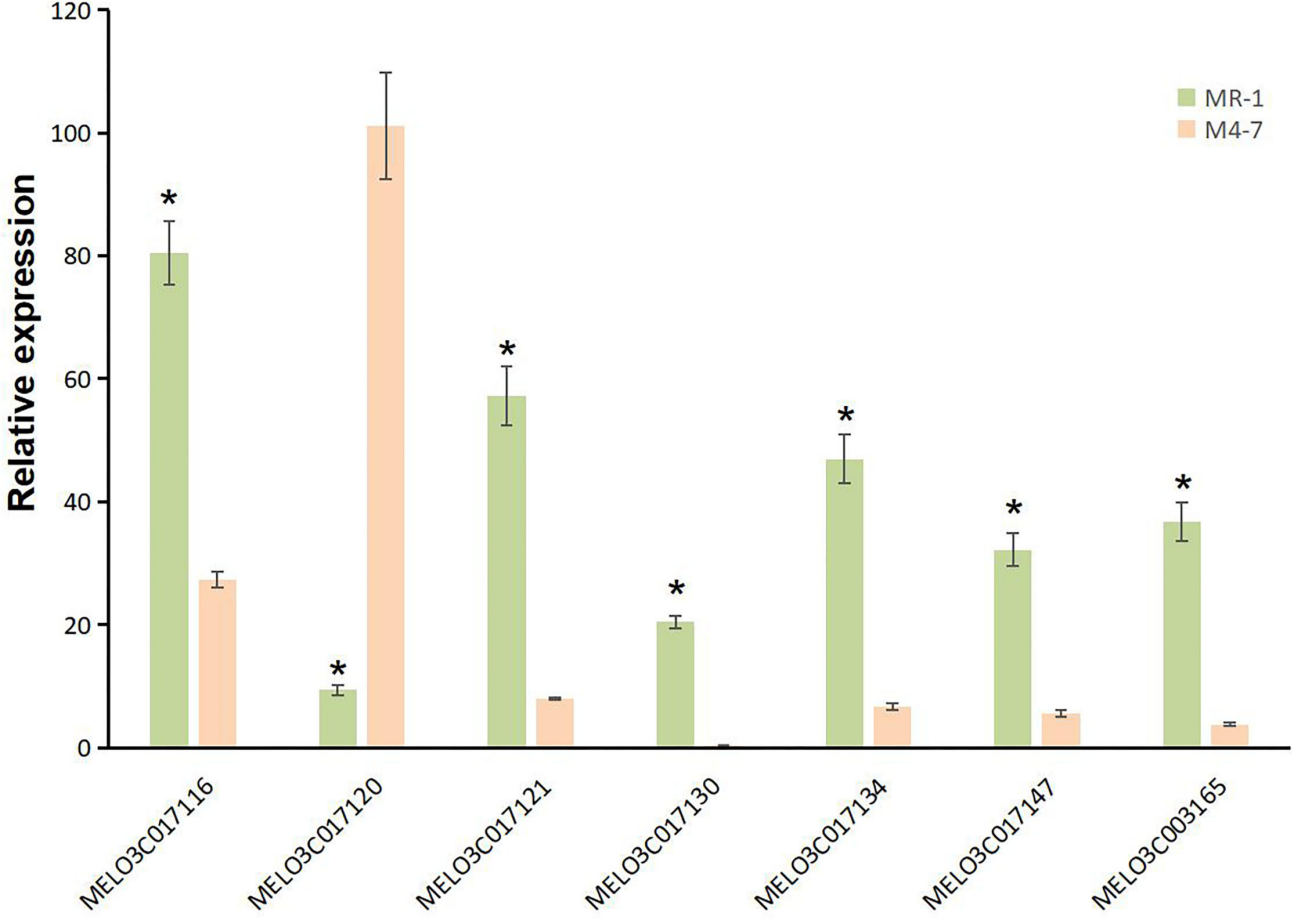
Expression analysis for candidate genes with non-synonymous single-nucleotide polymorphisms (SNPs) on chromosome 2 (*MELO03C017116, MELO03C017120, MELO03C017121, MELO03C017130, MELO03C017134*, and *MELO03C017147*) and chromosome 8 (*MELO03C017165*). * represents the presence of significant differences.

A total of 10 and 26 genes with non-synonymous SNPs (nsSNPs) were identified as putative genes positioned on chromosomes 2 and 8 (Tables 5, 6), and qRT-PCR was performed to identify the potential genes controlling stigma color, respectively (Supplementary Figure S1). A single gene (*MELO03C003165*) exhibited the significant differential expression profiling between parental lines at a 138 kb CI of *SC8.1* (Figure 7). In addition, a total of 6 genes exhibited significant differential expressions at a 180 kb CI of *SC2.1*.

**Table 5:** Predicted 26 genes with non-synonymous single-nucleotide polymorphisms (SNPs) between markers S2M3 and S2B1-2.

**Gene ID**	**nsSNPs**	**Gene annotation**
*MELO3C017116*	2	Kinesin-like protein
*MELO3C017117*	1	Calmodulin-binding protein 60 A-like isoform X1
*MELO3C017120*	2	Peroxidase 41-like
*MELO3C017121*	3	RING-type E3 ubiquitin transferase
*MELO3C017123*	6	Alpha-n-acetylglucosaminidase, putative
*MELO3C017124*	2	CDP-diacylglycerol-glycerol-3-phosphate 3-phosphatidyltransferase, putative
*MELO3C017125*	1	Betaine-aldehyde dehydrogenase
*MELO3C017126*	1	Mannose-1-phosphate guanyltransferase, putative
*MELO3C017127*	2	E2F transcription factor-like E2FE
*MELO3C017128*	1	Two-component response regulator ARR5-like
*MELO3C017129*	1	Chalcone synthase
*MELO3C017130*	3	Enhanced disease susceptibility 1
*MELO3C017132*	3	Microtubule-associated protein TORTIFOLIA1
*MELO3C017134*	1	Transcription factor MYB1R1
*MELO3C017135*	1	Serine/threonine-protein kinase AtPK2/AtPK19-like
*MELO3C017136*	1	Transportin-3
*MELO3C017138*	4	Germin-like protein subfamily T member 2
*MELO3C017141*	4	Dynein light chain
*MELO3C017143*	2	Transcription factor MYB35-like
*MELO3C017144*	1	DDT domain-containing protein DDR4
*MELO3C017145*	2	Protein NUCLEAR FUSION DEFECTIVE 4-like
*MELO3C017146*	2	Protein NUCLEAR FUSION DEFECTIVE 4
*MELO3C017147*	9	Zinc finger protein, putative
*MELO3C017148*	4	Ubiquitin carboxyl-terminal hydrolase-related protein
*MELO3C017149*	2	Pentatricopeptide repeat-containing protein
*MELO3C017152*	2	Protein NRT1/PTR FAMILY 2.10-like

**Table 6:** Predicted 10 genes with non-synonymous single-nucleotide polymorphisms (SNPs) between markers S8P19 and S8H9.

**Gene ID**	**nsSNPs**	**Gene annotation**
*MELO03C003159*	2	Cysteine/Histidine-rich C1 domain family protein, putative
*MELO03C003160*	1	Cysteine/Histidine-rich C1 domain family protein, putative
*MELO03C003161*	4	Cysteine/Histidine-rich C1 domain family protein, putative
*MELO03C003162*	1	Early nodulin-75-like
*MELO03C003163*	1	Cysteine/Histidine-rich C1 domain family protein, putative
*MELO03C003164*	2	Cysteine/Histidine-rich C1 domain family protein, putative
*MELO03C003165*	1	Uracil phosphoribosyltransferase isoform X1
*MELO03C003167*	3	Glycine-rich cell wall structural protein 1.0-like
*MELO03C003169*	1	Ctenidin-1-like
*MELO03C003172*	1	Protein MIZU-KUSSEI 1

The new marker 8B-13 used in 2021 is derived from the major effective QTL *GS8.1* obtained from another set of mapping populations used in a previous report from our lab (Qiao et al., 2021). Interestingly, QTLs representing the similar positions were obtained from two different populations, leading us to conclude that *SC8.1* is a stable QTL, which could have a major contribution to the regulation of stigma color. The gene *MELO03C003165* located in *GS8.1* also displayed the same nsSNP mutation site (SNP^30, 467, 367^), which was located in the exon region at 30,467,367th bp position. In MR-1, a base encodes leucine (Leu), whereas this base is mutated as T in the M4-7 line and significantly resulted in an amino acid change from Leu to histidine (His). Therefore, we analyzed *MELO03C003165* alleles across 110 melon varieties with different stigma colors (40 yellow stigmas and 70 green stigmas) and similarly checked the relationship between alleles and phenotype differences (Supplementary Table S2). The results showed that 78% of green stigma varieties had the same genotype with MR-1 and 75% of yellow stigma varieties had the same genotype with M4-7 (Figure 6B).

## Discussion

The stigma color phenotype is often considered as a communication signal for natural pollinators of flowers, and its differential diversity is driven by the natural selection process. At the physiological level, pigment content accumulation and chloroplast development are associated with differential coloration in higher plants (Yang et al., 2015). Different concentrations of chlorophyll and carotenoid components were known to cause a variety of colors in cucurbit fruits (Henderson et al., 1998; Burger et al., 2010; Zhang et al., 2016).

Development and usage of bi-parental F_2_ population are known as a conventional and rapid method to identify the significant putative genes regulating the important traits. The size of F_2_ mapping population can reduce the influence of environmental factors and reduce the biasness of accurate findings (Amanullah et al., 2021). In this study, we used fairly good mapping populations of F_2_ and F_2:3_ families and effectively identified two stable QTLs in chromosomes 2 and 8, harboring the potential genes associated with stigma color. Further, genetic mapping in the expanded F_2_ population also signified the delimited target regions of *SC2.1* and *SC8.1*, respectively.

The QTL *SC8.1* was consistent with the genetic position of major QTL *GS8.1* that is reported in the previous report of our lab (Qiao et al., 2021), which similarly suggested the conservation of genetic mechanisms governing stigma color across distinct germplasms in melon. The gene *MELO03C003165* was the only gene with nsSNP mutation locus in both candidate QTLs (*SC8.1* and *GS8.1*) (Table 6). However, this gene exhibited to encode the uracil phosphoribosyl transferase (UPRT), triggered the plastid levels (Mainguet et al., 2009). The UK/UPRT has a dual role in coding both uridine kinase and uracil phosphoribosyltransferase that form uridine 5′-monophosphate (UMP) through the pyrimidine salvage pathway in *Arabidopsis* and regulates chlorophyll content in plants (Islam et al., 2007; Mainguet et al., 2009).

Further, our RT-qPCR analysis revealed 6 candidate genes harboring putative involvement in stigma color and similarly have explicit description, e.g., *MELO03C017116, MELO03C017120, MELO03C017121, MELO03C017130, MELO03C017134*, and *MELO03C017147* (Table 5). The gene *MELO03C017116* encodes kinesin-like protein (KAC), which is required for chloroplast dispersion within the cell under standard culture conditions (Suetsugu et al., 2012). In addition, KACs mediate the chloroplast light evasion response in an actin dependent (Shen et al., 2015). The gene *MELO03C017120* encodes peroxidase, which mediates chlorophyll degradation in the chloroplast or vacuole in the presence of phenolic compounds, such as apigenin (Yamauchi et al., 2004). The gene *MELO03C017121* encodes ring-type E3 ubiquitin transferase, which is homologous to the rice *GW2* gene encoding ring-type E3 ubiquitin transferase. The earlier studies have confirmed that *OsGW2* controlled the chlorophyll content with positive regulation of leaf senescence through genetic analysis of a knockout mutant (Shim et al., 2020). The gene *MELO03C017130* encodes enhanced disease susceptibility 1 (EDS1), which has been shown to affect many biological processes, such as chlorophyll content and reactive oxygen species (*ROS*) metabolism in annual plants (*Arabidopsis thaliana*) and woody plants (*Populus tremula* L. × *P*. *tremuloides*) (Su et al., 2014; Bernacki et al., 2018). The gene *MELO03C017134* encodes transcription factor *MYBR1*, and loss-of-function in *Arabidopsis* plants showed more rapid chlorophyll loss and senescence (Jaradat et al., 2013). The gene *MELO03C017147* encodes zinc finger protein, and it has been reported that overexpression of zinc-finger protein gene (such as *RHL41, AtZFP1*) resulted in an increase in chlorophyll content of *Arabidopsis* (Kazuoka et al., 2010; Han et al., 2014). *PSA2* is a member of the DnaJ-like zinc finger domain protein family that affects the light acclimation and chloroplast development (Wang et al., 2016). In conclusion, further screening of our candidate genes using more rigorous methodologies will provide more light in this direction.

At present, we infer that the difference in the stigma color of melon might be caused by the difference in total chlorophyll content in accordance with our results. There are other studies with similar results, chlorophyll deficiency in mutant tomato fruits triggered the yellowish skin color due to abnormal chloroplast development (Liu et al., 2021). At higher latitudes, some cucumbers are naturally subjected to a longer time for photosynthesis and chlorophyll synthesis, which results in green flesh color (Bo et al., 2019). However, the involvement of candidate genes in the synthesis and degradation of chlorophyll similarly indicate the specific biological pathway responsible for the differences in color of melon stigma, which need to be further investigated.

In addition, our field observations confirmed this favored behavior in bees feeding on melon flowers and showed a preference to visit the flowers with green stigma than flowers with yellow stigma. This confirms beyond the doubt that green stigmas are of greater value to melons both for farming and breeding programs. Thus, novel outcomes would be beneficial to provide the fundamental basis for the in-depth genetic understanding of the melon stigma color traits.

## Data Availability Statement

The original contributions presented in the study are included in the article/Supplementary Material, further inquiries can be directed to the corresponding author.

## Author Contributions

YL performed the experiment, data curation, formal analysis, manuscript draft, and reviewed and edited the manuscript. PG and SL guided for the theoretical and practical experiments. XF, TZ, TL, and XW helped in formal analysis. SA reviewed and edited the manuscript language. FL supervised the research project and reviewed and edited the manuscript. All authors approved the final version of manuscript and disclosed no conflicts of interest.

## Funding

This work was supported by the National Nature Science Foundation of China (31772331 and 32030094), the China Agriculture Research System of MOF and MARA (CARS-25), and Taishan Industrial Leading Talents Project [grant no. LJNY202112].

## Conflict of Interest

The authors declare that the research was conducted in the absence of any commercial or financial relationships that could be construed as a potential conflict of interest.

## Publisher's Note

All claims expressed in this article are solely those of the authors and do not necessarily represent those of their affiliated organizations, or those of the publisher, the editors and the reviewers. Any product that may be evaluated in this article, or claim that may be made by its manufacturer, is not guaranteed or endorsed by the publisher.
