# Supplementary material for: Genetic Mapping and QTL Analysis of Stigma Color in Melon (Cucumis melo L.)
Source: Front Plant Sci. 2022 May 9;13:865082. doi: 10.3389/fpls.2022.865082 (PMC9125322; doi:10.3389/fpls.2022.865082)
Supplement: Supplementary file 1 [file Data_Sheet_1.zip › Supplementary Figure S1.docx]

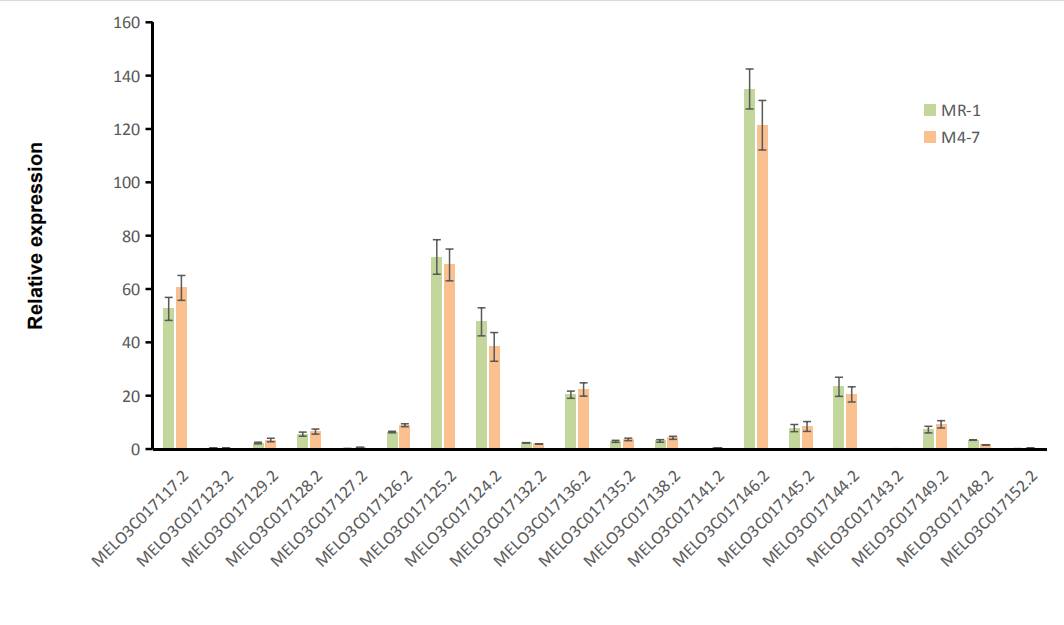


SUPPLEMENTARY FIGURE 1 | Expression analysis for predicted genes with nonsynonymous SNPs on chromosome 2
