# Supplementary material for: Genetic Mapping and QTL Analysis of Stigma Color in Melon (Cucumis melo L.)
Source: Front Plant Sci. 2022 May 9;13:865082. doi: 10.3389/fpls.2022.865082 (PMC9125322; doi:10.3389/fpls.2022.865082)

SUPPLEMENTARY TABLE 1 | The significance differences in L, A, B and E between parents and F_2_ by using ANOVA.


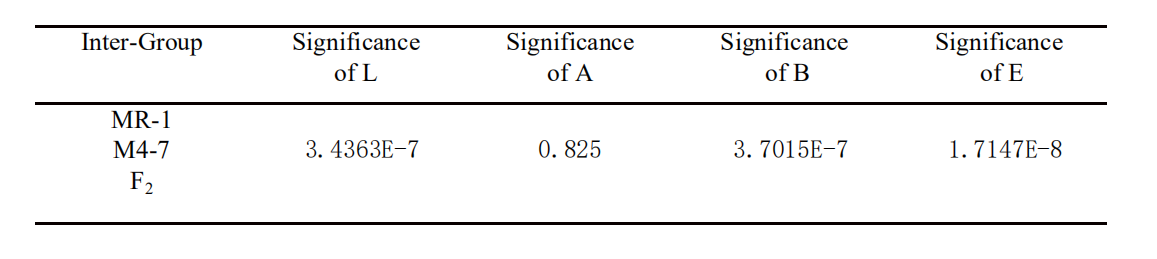

Supplement: Supplementary file 1 [file Data_Sheet_1.zip › Supplementary Table S1.docx]
